# Hyperuricemia in heart failure with preserved ejection fraction: pathophysiological insights and therapeutic perspectives – a narrative review

**DOI:** 10.1097/MS9.0000000000005130

**Published:** 2026-05-20

**Authors:** Manahil Riaz, Aakash Ghanwani, Maira Khan, Vishaka Sahjwani, Varoon Kumar, Umber Fatima Farooqui, Hina Gul, Maliha Said, Sofia Shah, Muhammad Saeed Qazi, Mohammed Hammad Jaber Amin, Syed Sameer Naqvi

**Affiliations:** a Department of Medicine, Ayub Medical College, Abbottabad, Pakistan; b Department of Medicine, Ziauddin Medical College, Karachi, Pakistan; c Department of Medicine, Karachi Medical and Dental College, Karachi, Pakistan; d Department of Medicine, Dow International Medical College Karachi, Pakistan; e Department of Medicine, Khyber Girls Medical College, Peshawar, Pakistan; f Department of Medicine, Saidu Medical College, Swat, Pakistan; g Department of Medicine, Bilawal Medical College, Liaquat University of Medical and Health Sciences, Jamshoro, Pakistan; h Department of Medicine Alzaiem Alazhari University, Khartoum, Sudan

**Keywords:** heart failure with preserved ejection fraction, hyperuricemia, prevalence, treatment

## Abstract

Heart failure with preserved ejection fraction (HFpEF) constitutes a growing burden worldwide, with hyperuricemia emerging as a prominent comorbidity. This review explores the prevalence, pathophysiological mechanisms, and therapeutic implications of hyperuricemia in HFpEF. Epidemiological studies consistently demonstrate that elevated serum uric acid (SUA) levels are associated with an increased risk of cardiovascular events, hospitalizations, and mortality. Mechanistic insights highlight endothelial dysfunction, oxidative stress, myocardial fibrosis, and systemic inflammation as key contributors linking hyperuricemia to adverse cardiac remodeling. Pharmacological interventions, including xanthine oxidase inhibitors, uricosuric agents, and SGLT2 inhibitors, show promise in mitigating hyperuricemia and improving HFpEF outcomes. Despite emerging evidence, critical knowledge gaps persist regarding causality, therapeutic efficacy, and optimal patient selection. Future research should focus on targeted interventions, dynamic SUA monitoring, and the interplay between hyperuricemia, diabetes, and left ventricular dysfunction to enhance the management of HFpEF patients.

## Introduction

Heart failure with preserved ejection fraction (HFpEF) is a complex multi-organ syndrome that affects around half of all patients with heart failure (HF) and has become a major public health concern, especially as its prevalence is growing at a startling rate^[^1^]^. The rate of incidence is estimated to be approximately 27 cases per 10 000 person-years across four community-based cohorts^[^2^]^. For both men and women, the predicted lifetime risk of HFpEF at age 45 is greater than 10%^[^3^]^. Moreover, over a 5-year period, HFpEF has significantly high hospitalization and mortality rates, up to 80 and 50%, respectively, across all demographics^[^4^]^.

HIGHLIGHTS

- Hyperuricemia is a key prognostic marker in heart failure with preserved ejection fraction (HFpEF) progression and outcomes.
- An elevated uric acid level drives endothelial dysfunction, oxidative stress, and myocardial fibrosis.
- Xo and SGLT2 inhibitors show potential for reducing uric acid in HFpEF.
- Managing hyperuricemia may improve HFpEF morbidity and mortality.
- Large randomized trials are needed to validate the benefits of uric acid reduction therapies.

The increase in prevalence can be attributed to the growing number of patients with non-cardiac comorbidities of HFpEF, including diabetes mellitus (DM), hypertension (HTN), obesity, anemia, chronic kidney disease (CKD), chronic obstructive pulmonary disease (COPD), and hyperuricemia. All of these lead to a systemic pro-inflammatory state^[^5^]^. It was observed that serum C-reactive protein (CRP) concentration rises with the increasing number of comorbidities^[^6^]^. In response to systemic inflammation, endothelial cells of the coronary vasculature undergo certain modifications that ultimately increase stiffness and cause fibrosis of the myocardium^[^7^]^. This results in the development of a stiffened, hypertrophied left ventricle (LV) and diastolic dysfunction^[^8^]^. However, diastolic dysfunction is just one part of the puzzle that is HFpEF. In more recent years, other mechanisms have also been identified as key players in this highly complex disease, including left atrial myopathy, chronotropic incompetence, pulmonary hypertension, and abnormal vasodilation^[^9–11^]^.

Uric acid (UA), a byproduct of the body’s purine metabolism, is frequently linked to the development and advancement of cardiovascular disorders. Hyperuricemia is a common comorbidity of HF and is associated with a worse prognosis, imposing a significant clinical burden on patients by contributing to a higher risk of HF hospitalization and increased mortality^[^12,13^]^. Xanthine oxidase (XO) is the enzyme that catalyzes UA production and is a potent source of reactive oxygen species (ROS). Like a positive feedback cycle, the existing systemic inflammation caused by non-cardiac comorbidities leads to XO activation, which increases oxidative stress and further exacerbates the already present systemic inflammation^[^14,15^]^. This inflammatory condition results in structural and functional changes in the myocardium, with an increased predisposition to complications such as malignancies and infections, which contribute to higher mortality rates in HFpEF^[^2,16^]^. Furthermore, endothelial cells in the coronary vasculature are damaged, leading to a decreased vasodilator response to acetylcholine, which correlates with LV diastolic dysfunction^[^17^]^. Additionally, UA may aggravate HF directly by raising blood pressure and impairing renal function^[^18,19^]^.

Although progress has been made in our understanding of the epidemiology, pathophysiology, molecular mechanisms, diagnosis, and therapy of HFpEF over the past decade, significant knowledge gaps and unmet needs remain, especially regarding hyperuricemia. UA-lowering therapy (ULT) could have therapeutic potential beyond traditional HF medications, but there is limited research on it. In this review, we will examine the importance of UA-lowering drugs and the existing literature on the prevalence, cardiovascular profile, and management of hyperuricemia in patients with HFpEF.

In line with the TITAN 2025 guidelines^[^20^]^ on declaring AI usage in research and manuscript preparation, we affirm that no generative AI tools were employed in the conceptualization, drafting, or revision of this manuscript.

## Methodology

A comprehensive search strategy was applied across databases such as PubMed, Embase, Cochrane, Medline, and Google Scholar from inception to March 2025 to ensure the inclusion of relevant literature on the association between hyperuricemia and HFpEF. Boolean operators AND, OR, and NOT were used to create search strings employing keywords such as “hyperuricemia,” “HFpEF,” “cardiovascular phenotype,” and “treatment” to refine our search strategy. The reference lists of the selected literature were manually screened to broaden the scope of our search.

Exclusion and inclusion criteria were clearly defined. Peer-reviewed full-text articles, abstracts, and papers available in English were included. Exclusion criteria encompassed non–peer-reviewed articles, retracted and unpublished literature, and articles that did not sufficiently discuss our topic.

## Prevalence of hyperuricemia in HFpEF

### Epidemiology

UA is the end result of purine metabolism, which occurs largely in the liver, muscles, and intestines. Hyperuricemia is diagnosed when serum UA (SUA) levels surpass 7 mg/dL for men and 6 mg/dL for women. This causes UA crystals to precipitate in the blood^[^21^]^. In various populations, the prevalence of hyperuricemia varies from 13.4 to 20.1%^[^22–24^]^.

The growing proportion of HFpEF compared to HF with reduced ejection fraction (HFrEF) has been identified as a public health concern^[^25,26^]^. The mortality rates were similar between patients with HFpEF and those with HFrEF^[^27^]^.

Increased UA levels are not only the cause of gout, but they may also play a role in the development of cardiovascular diseases (CVD)^[^28,29^]^. Although hyperuricemia is prevalent in patients with chronic HF (with a prevalence of 50%)^[^30^]^, it is only 26% in HFpEF^[^11^]^, according to the European Society of Cardiology’s (ESC) guidelines, which emphasize UA testing as an additional marker for the classification of cardiovascular risk^[^31^]^. Furthermore, hyperuricemia has emerged as a prominent comorbidity of HF. Recent research strongly indicates that hyperuricemia plays a crucial role in increasing cardiovascular risk^[^32–34^]^. A systematic review and meta-analysis examining HF morbidity and outcomes in adult patients found that hyperuricemia was linked to a higher risk of incident HF [hazard ratio (HR): 1.65, 95% confidence interval (CI): 1.41–1.94], and the odds of developing HF rose by 19% for every 1 mg/dL increase in UA^[^13^]^. Another meta-analysis states that every 1 mg/dL reduction in UA is associated with significantly decreased cardiovascular death and hospitalization due to HF^[^35^]^.

### Comorbidities

The cause of HFpEF is still not fully known, but it is believed to be influenced by underlying systemic pro-inflammatory diseases like obesity, DM, HTN, and metabolic syndrome^[^36^]^. In another study, subjects with hyperuricemia and HFpEF also had DM (34%), HTN (64%), Coronary Artery Disease (CAD) (29%), atrial fibrillation (48%), and CKD (39%)^[^37^]^.

Multiple observational studies report similar comorbidity clusters among HFpEF patients with hyperuricemia, as shown in Table 1. Numerous epidemiologic studies have looked into the relationship between gout or hyperuricemia and the risk of CVDs^[^38–41^]^. Specifically, hyperuricemia has been associated with an increased risk of any^[^39^]^ and specific subtypes of CVD, including stroke^[^38^]^, CAD^[^42^]^, incident HTN^[^39^]^, atherosclerosis^[^43^]^, and atrial fibrillation^[^44^]^. Likewise, with a further enhanced magnitude of association, gout was noted as an independent risk factor for CAD, peripheral arterial disease, HF, stroke, and CVD mortality, suggesting a continuum of an increase in CVD risk from hyperuricemia to gout^[^40–48^]^.

**Table 1:** Comorbidities of hyperuricemia in HFpEF studies.

Year	Total sample size/hyperuricemia patient	Uric acid level (mean ± SD)	Comorbidities
Liu *et al*, 2023	538/310	8.5 ± 1.8	Hypertension (68.4%), diabetes mellitus (32.1%), coronary artery disease (27.5%), atrial fibrillation (15.8%)
Shimizu *et al*, 2015	424/254	7.1 ± 1.2	Hypertension 203 (79.9), Diabetes,98 (38.5), Dyslipidemia 203 (79.9), Atrial fibrillation 123 (48.4), CKD 171 (67.3), Anemia 169 (66.5)
Palazzuoli *et al*, 2017	331/80	>7.0 mg/dL	Diabetes mellitus 34%, hypertension 64%, CAD 29%, atrial fibrillation 48%, CKD 39%
Liang Deng *et al*, 2023	210/80	>7.0 mg/dL	Association with right ventricular dysfunction

Previous research has demonstrated a link between increased SUA and a higher prevalence of cardiovascular events, such as HTN, atrial fibrillation, myocardial infarction, acute coronary syndrome, and CAD. Increased UA levels may also lead to worse prognoses in patients with HF related to acute coronary syndrome and are a strong marker for prognosis and severity^[^49^]^. High serum urate levels are also strongly associated with metabolic syndrome, which is a cluster of cardiovascular risk factors that carries an increased risk of future cardiovascular events. Levels higher than 7 mg/dL and 6 mg/dL in men and women, respectively, lead to a 5.13-fold increase in the risk of developing metabolic syndrome. With URRAH’s cut-off values for UA, that risk is 3.53-fold. The greater the value of UA, the stronger the relationship between serum urate and metabolic syndrome^[^50^]^.

As for the cut-off value, an analysis of the URRAH study results identified 5.6 mg/dL as the cut-off for cardiovascular mortality, and 5.34 mg/dL and 4.89 mg/dL for HF and fatal HF, respectively^[^51^]^. Other studies report different, heterogeneous cut-offs for prognosis, with sex differences having an effect as well; for males, the value was 7.76 mg/dL, lower than 9.26 mg/dL for females in one study^[^52^]^. Another study analyzing patients with an acute MI and HFpEF found that the optimal SUA cut-off was >6.9 mg/dL in men and >5.4 mg/dL in women^[^53^]^. Moreover, mortality due to coronary heart disease increased by 12% with a 1 mg/dL increase in the SUA level^[^1,54–59^]^.

## Cardiovascular phenotype and pathophysiology

### Mechanism of UA elevation

Etiologically, hyperuricemia depends on genetic predisposition, environmental factors, and complicated metabolic pathways that interfere with UA homeostasis. Studies have shown that hyperuricemia can occur due to high intake of purine-rich sources [animal protein (meat and seafood) and beer], fructose-rich foods, alcohol consumption, increased cell turnover (e.g., hemolysis, tumor growth, and large tumor necrosis), genetic variants, and diseases such as obesity, DM, CKD.^[^60–64^]^

Hyperuricemia is a common finding in patients with HFpEF^[^65^]^. Experimental and clinical studies have suggested hyperuricemia as a bridging mechanism, culminating in deleterious effects in HF. The following are components of the pathophysiological mechanisms that link hyperuricemia to HFpEF.

#### Endothelial stress

Endothelial cells secrete both vasodilators and vasoconstricting substances. Nitric oxide (NO), prostaglandin E2, and the endothelium-derived hyperpolarizing factor are some of the vasodilators secreted by endothelial cells^[^66^]^. NO, which plays an important role in atherosclerosis, combines with excess UA in the cell. Hence, less NO is available to the cell, and the levels of peroxynitrite (ONOO^−^), a strong oxidant, increase^[^67^]^. This causes lipid peroxidation, DNA damage, and cell death^[^68^]^. Additionally, it is found that an elevated UA level blocks endothelial nitric oxide synthase expression and the production of NO in human umbilical vein endothelial cells (HUVECs). High mobility group box chromosomal protein 1, receptor for advanced glycation end products (RAGE), activates Nuclear Factor-kappa B (NF-κB) and cytokines^[^67,69^]^. All these factors contribute to endothelial dysfunction with high UA levels.

#### Oxidative stress

XO contributes to oxidative stress in HF by producing UA and ROS. UA may function as both an antioxidant and a pro-oxidant in a hydrophobic intracellular environment by forming free radicals or NADPH oxidase^[^69^]^. Unlike UA, XO-derived ROS promotes oxidative stress and is presumed to have a notable effect on cardiac function in higher mammals, contributing to myocardial failure. ROS interferes with endothelial-derived NO, which produces ONOO^−^, initiates a complex oxygen radical cascade, and creates deleterious effects on endothelial cells. High UA levels and ROS induce oxidative stress, lipid peroxidation, mutagenesis of the cell’s genetic material, and irreversible damage to cardiomyocytes. There is a study that describes the role of the renin-angiotensin-aldosterone system (RAAS) in UA-induced oxidative stress and endothelial function^[^70^]^. ROS leads to remodeling of cardiac tissues in chronic HF by promoting cardiac fibroblast proliferation and activating XO-induced matrix metalloproteinase (MMP)^[^70–73^]^ Activation of MMP causes myofibrillar degeneration and myosin-troponin degradation during the course of ischemic/reperfusion injury of the heart, resulting in left ventricular (LV) dysfunction^[^73^]^. According to a study by James *et al*, ROS decreases the contractility of the heart by depressing calcium ion (Ca^+2^) accumulation and Ca^+2^ ATPase activity in the sarcoplasmic reticulum^[^74^]^. In conclusion, oxidative stress plays a key role in the pathogenesis of HF with hyperuricemia, being implicated in myocardial fibrosis, LV and endothelial dysfunction, and disturbed contractility of cardiomyocytes. All of this culminates in a worse prognosis for HF.

#### Left ventricular dysfunction

LV dilation and hypertrophic myocardial dysfunction can be found as a decompensatory cardiac function mechanism in patients with HF, with decreased ventricular compliance and systolic function contributing to the HF. Studies suggest that it contributes to echocardiographic changes that lead to vascular dysfunction and inflammation in HF. Deposition of collagen restricts the ability of the myocardium to contract and relax, leading to HFpEF fraction^[^75,76^]^. Moreover, hyperuricemia activates Calpain-1, endoplasmic reticulum stress, and ROS-dependent endothelin-1 (ET-1) pathways^[^75–78^]^. These mechanisms are responsible for inducing cardiomyocyte apoptosis, interstitial fibrosis, diastolic dysfunction, and remodeling.

#### Vascular inflammation

Inflammation through node-like receptor protein-3 (NLRP3), inflammasome-mediated vascular smooth muscle cell (VSMC) proliferation, adenosine monophosphate (AMP)–mediated protein kinase, and NF-κB are important pathways for vascular inflammatory response in hyperuricemia^[^77–79^]^. Chemokines and adhesive molecules released by UA form an endothelial cell tube in HUVECs in a dose-dependent manner^[^79^]^. UA and monosodium urate lead to the formation of ROS and mediate inflammation, which in turn leads to HF.

#### Left ventricular mass index

LV hypertrophy (LVH) in patients with elevated UA levels or hyperuricemia is highly prevalent, representing another risk factor for cardiovascular and cardiovascular-related events^[^80^]^. Comorbidities such as diabetes mellitus, obesity, and hypertension are also common in patients with hyperuricemia, which further contribute to LVH progression. Using a cut-off of 5.6 mg/dL, Visco *et al* demonstrated a direct correlation between high serum urate levels and greater LV mass in patients^[^81^]^. Due to the systemic pro-inflammatory state, elevated levels of C-reactive protein, tumor necrosis factor α (TNFα), interleukin-6 (IL-6), growth differentiation factor 15 (GDF15), interleukin-1 (IL-1) receptor 1, and IL-1 receptor-like 1 result in microvascular endothelial dysfunction, myocardial immune cell infiltration, and destabilized protein accumulation. All of this leads to greater cardiac mass, LVH, myocardial stiffness, and diastolic dysfunction^[^8,82,83^]^. UA has been hypothesized to affect diastolic dysfunction during its early stages, but later it is overshadowed by other factors^[^84^]^. More testing and new research are required for a definitive answer.

#### Usage of diuretics

Medications such as loop and thiazide diuretics are known to cause iatrogenic hyperuricemia^[^85^]^, increasing serum urate levels in a dose-dependent manner^[^86^]^. They have been shown to increase UA levels by 0.3–1.1 mg/dL above baseline after a 10-week treatment^[^87,88^]^. They enter proximal tubular cells via organic anion transporters, then exit into the lumen via organic anion transporter 4, reabsorbing UA in their place^[^89–92^]^. This diuretic-related hyperuricemia has the same risk profile as hyperuricemia without diuretics^[^93^]^.

### Hyperuricemia and its role in the pathophysiology of HFpEF

Studies have suggested that increased UA levels are associated with cardiovascular mechanisms that result in the progression and severity of HF. These mechanisms include low cardiac output, increased filling pressure, heart muscle remodeling, elevated levels of B-type natriuretic peptide (BNP), and endothelial dysfunction. Moreover, elevated SUA in patients with HFpEF can lead to gout^[^79^]^. According to URRAH, a study in Italy with 22 714 participants, there is an increased risk of cardiovascular mortality for people with SUA levels greater than 5.6 mg/dl^[^94^]^. Some studies suggest hyperuricemia as a predictor of rehospitalization and all-cause death in patients with HFpEF^[^95^]^.

### Relationship with comorbidities:

There is a profound relationship between insulin resistance or Type 2 diabetes mellitus (T2DM) and hyperuricemia. It is said that one-fourth of all diabetic patients have elevated UA levels^[^96^]^. Hyperuricemia is correlated with albuminuria and retinopathy in patients with non-insulin-dependent DM^[^97^]^. Moreover, high UA levels cause overgeneration of ROS, which leads to oxidative stress and subsequently to insulin resistance in patients with disturbed cardiac activity. Hyperuricemia blocks insulin-dependent glucose uptake receptors in H9c2 cells and cardiomyocytes. On a molecular basis, high UA levels increase the phosphorylation of insulin receptor substrate 1 (IRS1) and restrict the phosphorylation of Akt, which was initially blocked by N-acetyl L-cysteine^[^98^]^. Hence, hyperuricemia causes insulin resistance in myocardial cells. In general, the mechanism of hyperuricemia-induced insulin resistance in HF remains controversial and has not been fully elucidated to date^[^99^]^.

## Treatment options for hyperuricemia in HFpEF

Techniques for reducing UA levels in HFpEF aim to balance metabolic and cardiovascular factors. Effective strategies include a low-purine diet, exercise, weight loss, and hydration. Allopurinol and febuxostat are examples of xanthine oxidase inhibitors (XOIs) that might lower UA and enhance endothelial function; however, uricosurics like probenecid should be used with caution in patients with renal impairment. Emerging research suggests that sodium–glucose co-transporter-2 (SGLT-2) inhibitors can help lower UA levels and improve HFpEF outcomes. Diuretic optimization, particularly with loop and thiazide diuretics, is critical for managing fluid status without worsening hyperuricemia. Furthermore, managing comorbidities such as HTN, DM, and obesity helps to reduce metabolic stress. Regular monitoring is necessary to achieve adequate UA management while not exacerbating HFpEF symptoms.

### Pharmacological interventions

Several pharmacological agents have been investigated for managing hyperuricemia in HFpEF, including verinurad, allopurinol, empagliflozin, benzbromarone, and spironolactone. Their mechanisms of action, efficacy, and adverse effects are summarized in Table 2.

**Table 2:** Drugs for hyperuricemia and HFpEF: mechanisms, efficacy, and adverse effects.

Drug	Mechanism of action	Efficacy	Adverse effects
Verinurad	Selective URAT1 inhibitor that increases the excretion of uric acid by decreasing its reabsorption in the kidneys	Effectively reduces blood uric acid levels, showing promising results in gout and hyperuricemia patients.	Risk of renal impairment, particularly in those with pre-existing kidney disease.
Allopurinol	Xanthine oxidase inhibitor, decreasing uric acid production by inhibiting the enzyme responsible for its synthesis.	It decreases serum uric acid levels, widely used for gout and hyperuricemia management.	Rash, gastrointestinal issues, liver toxicity, risk of hypersensitivity reactions, potential renal toxicity.
Empagliflozin	SGLT2 inhibitor reduces serum uric acid via increasing uric acid excretion from the kidneys.	Decreases serum uric acid levels and improves cardiovascular outcomes in HFpEF patients.	Dehydration, hypotension, urinary tract infections, potential for diabetic ketoacidosis.
Benzbromarone	Uricosuric agent that inhibits renal uric acid reabsorption, increasing its excretion in the urine.	Potent uric acid–lowering action, especially beneficial in patients with resistant hyperuricemia.	Hepatotoxicity (liver issues), gastrointestinal disturbances, contraindicated in liver disease.
Spironolactone	Aldosterone antagonist that helps reduce fluid retention and may indirectly affect uric acid excretion through its diuretic effect.	Can improve heart failure outcomes in HFpEF but has limited direct efficacy in lowering uric acid.	Hyperkalemia, renal dysfunction, gynecomastia, fatigue, dizziness.

Verinurad is a selective URAT1 inhibitor that increases the excretion of UA by reducing its renal reabsorption. This treatment for hyperuricemia is effective, as clinical trials show dose-dependent decreases in SUA levels^[^100^]^. SUA levels were significantly reduced in one experiment by both single and repeated once-daily doses of verinurad, which ranged from 1 to 40 mg^[^100^]^. According to these findings, verinurad shows promising results as a ULT for hyperuricemia and associated conditions.

Allopurinol is an XO inhibitor that helps hyperuricemic patients with CKD by reducing the formation of UA and maintaining renal function for 1 year^[^101^]^. Insulin resistance, systemic inflammation, and renal function are all improved with prolonged use (3.4 years on average)^[^102,103^]^. At higher doses, allopurinol also reduces the risk of cardiovascular events^[^104,105^]^.

Allopurinol and verinurad together may have a synergistic impact in lowering SUA levels in gout patients. A study evaluated verinurad’s pharmacokinetics, pharmacodynamics, and tolerability when combined with allopurinol. Verinurad was found to raise allopurinol’s maximum measured plasma concentration (C-max) by 33%, but it had no effect on the area under the plasma concentration–time curve (AUC). However, verinurad reduced the C-max and AUC of oxypurinol, which is allopurinol’s active metabolite, by 32% and 38%, respectively. Despite these pharmacokinetic interactions, the combination therapy achieved a maximum reduction in SUA (65%) compared to either allopurinol (43%) or verinurad (51%). When compared to either medication alone, the combination produced a larger decrease in SUA, and it was well tolerated^[^106^]^.

A multicenter randomized controlled trial comparing febuxostat to allopurinol in patients with chronic HF found that febuxostat, a selective XO inhibitor, was not superior to allopurinol. UA levels in both groups were reduced to a value below 6 mg/dL, but after dividing the population according to LV ejection fraction (LVEF), patients in the HFpEF group showed a non-significant difference in UA levels, whether they were given allopurinol or febuxostat (4.77 ± 1.27 vs 4.99 ± 0.97 mg/dL)^[^107^]^. A similar finding was demonstrated in the CARES trial, a multicenter, randomized, double-blind, non-inferiority study^[^108^]^, which also showed higher all-cause and CV mortality in patients taking febuxostat. However, due to the lack of a control, one cannot say if this is due to allopurinol’s protective effect or febuxostat’s negative effect^[134]^. However, the FREED trial, a multicenter, prospective, randomized, open-label, blinded-endpoint study in Japan, found a reduction in cardiovascular, renal, and cerebral events, and significantly reduced UA levels compared with conventional therapy and lifestyle modification. This may also lead to better prognosis in such patients as well. More large-scale trials are necessary to try to establish a link between hypouricemic drugs, with a focus on their effect on HF.

### Lifestyle changes

In addition to pharmacological interventions, certain lifestyle changes may help reduce UA levels, thereby slowing disease progression. According to the OmniHeart trial, a DASH-style diet with plant-based protein as the macronutrient reduced serum urate levels from baseline when compared to a DASH-style diet with carbohydrates or unsaturated fats as the macronutrient^[^109^]^. Ingestion of dairy products, such as milk, has an acute lowering effect on UA levels, as seen in a randomized control trial, where participants who ingested milk saw their levels drop by 10%^[^110^]^. A similar finding was observed in a survey assessing dietary factors and their effects on serum urate levels, where meat and seafood increased levels by 0.48 and 0.16 mg/dL, respectively, and total dairy intake reduced levels by 0.21 mg/dL^[^111^]^. Alongside dietary changes, weight loss from moderate calorie/carbohydrate restriction has shown a decrease in UA levels as well, in a pilot study^[^112^]^. A secondary analysis of the Dietary Intervention Randomized Control Trial (DIRECT), where participants lost weight over 6 months on three different diets, demonstrated a reduction in UA levels: −48 μmol/L (95% CI, − 65 to −24) for the low-fat diet, −48 μmol/L (−65 to −30) for the Mediterranean diet, and −48 μmol/L (−71 to −30) for the low-carbohydrate diet^[^113^]^. A clinical study showed a reduction of 0.3–3.2 mg/100 mL with chronic exercise in athletic and training groups, a significant decrease (*P* < 0.05) as compared to the sedentary control group^[^114^]^. However, more studies are needed to fully explore the effect of exercise solely on UA levels.

### Emerging therapies

Empagliflozin is an SGLT2 inhibitor that improves cardiovascular and renal outcomes, as well as lowers Systolic Blood Pressure (SBP) and UA levels in patients with HFpEF. Its effectiveness in HF patients was demonstrated by the EMPEROR-Reduced trial, regardless of their baseline BP^[^115^]^. A meta-analysis of 12 randomized controlled studies revealed that empagliflozin significantly decreased blood pressure, body weight, and UA levels in patients with type 2 diabetes (DMT2)^[^116^]^.

A study in Diabetes Care assessed the effectiveness, safety, and tolerability of empagliflozin in individuals with both DMT2 and HTN. Compared with placebo, the results showed a significant reduction in mean 24-hour SBP. For example, the mean 24-hour SBP change from baseline was −3.44 mm Hg for the 10 mg dose and −4.16 mm Hg for the 25 mg dose (both *P*-values < 0.001). These decreases were accomplished without raising heart rate^[^117^]^. It also improved hemoglobin and BNP levels in HFpEF patients, contributing to better health outcomes^[^118^]^.

Benzbromarone, a uricosuric drug, works by preventing renal reabsorption to efficiently reduce SUA levels. It is an effective treatment for gout because it has shown quicker lowering of UA levels than other medications^[^119^]^.

Spironolactone is an aldosterone antagonist that lowers hospitalizations for HF but may deteriorate renal function, especially in patients with advanced CKD. Risks, including hyperkalemia and renal impairment, must be reduced with routine monitoring^[^120,121^]^.

## Discussion:

Hyperuricemia is a prevalent condition among HFpEF patients and is linked to various negative clinical outcomes^[^99^]^. It is more commonly observed in HFpEF compared to HFrEF, with one study showing that 57% of HFpEF patients had elevated UA levels^[^37^]^. The pathophysiological mechanism behind this link can be explained by the fact that increased UA production causes endothelial dysfunction and oxidative stress by decreasing the production of antioxidants like NO and increasing the production of pro-oxidants like peroxynitrite. This, along with ROS production by XO, is responsible for cardiac remodeling and fibrosis that eventually lead to LV dysfunction^[^67–70^]^. Therefore, this condition is associated with several cardiovascular risk factors and organ damage, including increased arterial stiffness and reduced exercise capacity^[^122^]^. Evidence suggests that lowering UA levels could improve the prognosis for HFpEF patients^[^122^]^. While UA lowering therapies (ULT), particularly those involving XO inhibition, are being investigated, their role in managing HF is still being explored^[^99^]^.

SUA is a cost-effective and widely applicable laboratory test with multiple clinical uses^[^99^]^. Monitoring UA levels in HFpEF patients during treatment is important, as effective management of hyperuricemia may improve patient outcomes^[^123^]^. Addressing comorbidities is also a key aspect of managing HFpEF. However, further research is needed to determine whether controlling hyperuricemia specifically improves HFpEF prognosis^[^123^]^. Targeted interventions, such as ULT with XO inhibition, show promise^[^99^]^. For every 1 mg/dL increase in UA, the likelihood of developing HF rises^[^99^]^. Lowering UA levels, including through ULT, could enhance outcomes for patients with hyperuricemia and HFpEF^[^124^]^.

Current research on hyperuricemia in HFpEF faces several challenges. Many studies are limited by small sample sizes and short follow-up periods, hindering the ability to draw firm conclusions^[^95^]^. Additionally, some studies do not track changes in UA levels over time or assess the impact of UA reduction on patient outcomes^[^125^]^. The lack of a comprehensive assessment of right ventricular dysfunction (RVD) further complicates understanding the effect of UA on RVD outcomes^[^125^]^. Furthermore, there has yet to be research examining the relationship between ULT and RV function in HFpEF patients^[^125^]^. Many studies are observational in nature, making it difficult to assess causality or the role of UA in the development and progression of HFpEF. Meta-analyses reveal significant variability across studies, particularly in the association between UA levels and HF hospitalizations^[^95^]^. Inconsistent findings also exist regarding the connection between elevated UA and all-cause mortality in HFpEF^[^125^]^. Moreover, the potential cardioprotective effects of ULT are still uncertain, with unclear evidence on whether the benefits are due to XO inhibition or simply a reduction in UA levels. Additionally, many studies focus on specific populations, such as non-Asian groups, which limits the generalizability of the results^[^95^]^.

Several key questions and areas for further research regarding hyperuricemia in HFpEF remain unresolved. One critical issue is whether hyperuricemia is simply a marker of poor prognosis or if it actively contributes to the development of HFpEF^[^125^]^. Additionally, it is unclear whether reducing UA levels can improve RV function in HFpEF patients, which requires targeted studies^[^125^]^. To date, no research has examined the relationship between ULT and RV function in this context. The cardioprotective effects of ULT, whether through XO inhibition or simply lowering UA levels, have not been definitively established. More research is also needed to assess the prognostic impact of dynamic changes in UA levels and to understand how UA affects RV dysfunction outcomes. Lastly, the role of UA in the onset and progression of HFpEF needs further investigation. Addressing these unanswered questions through additional studies could provide clarity on the role of hyperuricemia in HFpEF and open up potential therapeutic approaches to improve patient outcomes.

## Future directions

The clinical significance of hyperuricemia in HFpEF is highlighted by new data, which is driving continued research into targeted treatments. Empagliflozin effectively lowered SUA levels and related clinical outcomes, such as acute gout and gouty arthritis, by 38% in the EMPEROR-Preserved study. Similarly, another trial demonstrated how allopurinol and verinurad, a urate transporter-1 (URAT-1) inhibitor, may reduce SUA in individuals with HFpEF^[^126,127^]^.

On the other hand, a meta-analysis pointed out that ULTs were not beneficial for survival and might actually raise all-cause mortality in HF patients.

As new developments arise from research in different aspects of HFpEF treatment, future clinical guidelines may include routine serum UA monitoring. Individualized treatment strategies could then be incorporated accordingly, while weighing the possible advantages and disadvantages for optimal HFpEF management.

## Conclusion

Hyperuricemia is a common and clinically significant comorbidity in HFpEF, contributing to endothelial dysfunction, oxidative stress, myocardial fibrosis, and adverse clinical outcomes. However, it remains under-recognized and is not consistently addressed in treatment strategies. ULT, particularly XO inhibitors and SGLT2 inhibitors, shows promise but requires further validation through large, randomized clinical trials. Alongside pharmaceutical therapy, lifestyle changes, such as different diets and exercise, can reduce severity but require robust clinical trials to evaluate their long-term effects. Understanding the mechanistic interplay between hyperuricemia, DM, and LV dysfunction is crucial. Addressing hyperuricemia could offer an untapped therapeutic avenue to improve morbidity and mortality in HFpEF patients.

## Data Availability

All data relevant to the study are included in the article or uploaded as supplementary information. Additional data related to this study are available from the corresponding author upon reasonable request.
